# Target position and avoidance margin effects on path planning in obstacle avoidance

**DOI:** 10.1038/s41598-021-94638-y

**Published:** 2021-07-27

**Authors:** Mohammad R. Saeedpour-Parizi, Shirin E. Hassan, Ariful Azad, Kelly J. Baute, Tayebeh Baniasadi, John B. Shea

**Affiliations:** 1 Department of Kinesiology, School of Public Health, Indiana University Bloomington, 1025 E 7th Street, Bloomington, IN 47405 USA; 2 School of Optometry, Indiana University Bloomington, Bloomington, IN USA; 3 Department of Intelligent Systems Engineering, Luddy School of Informatics, Computing, and Engineering, Indiana University Bloomington, Bloomington, IN USA; 4 Splendid Earth Wellness LLC, Seymour, USA

**Keywords:** Cognitive neuroscience, Motivation, Motor control, Visual system

## Abstract

This study examined how people choose their path to a target, and the visual information they use for path planning. Participants avoided stepping outside an avoidance margin between a stationary obstacle and the edge of a walkway as they walked to a bookcase and picked up a target from different locations on a shelf. We provided an integrated explanation for path selection by combining avoidance margin, deviation angle, and distance to the obstacle. We found that the combination of right and left avoidance margins accounted for 26%, deviation angle accounted for 39%, and distance to the obstacle accounted for 35% of the variability in decisions about the direction taken to circumvent an obstacle on the way to a target. Gaze analysis findings showed that participants directed their gaze to minimize the uncertainty involved in successful task performance and that gaze sequence changed with obstacle location. In some cases, participants chose to circumvent the obstacle on a side for which the gaze time was shorter, and the path was longer than for the opposite side. Our results of a path selection judgment test showed that the threshold for participants abandoning their preferred side for circumventing the obstacle was a target location of 15 cm to the left of the bookcase shelf center.

## Introduction

When walking through the environment, we often take a path that circumvents obstacles on the way to our designated goal. Humans are capable of avoiding obstacles during gait to designated target locations. This requires the recognition of objects in the environment which might interfere with arrival at the target location. Safe avoidance strategies are characterized by appropriate path and speed selections which depend on the obstacle crossing angle and estimation of the minimum distance^1,2^. Baxter and Warren^1^ recently followed-up on earlier research by Fajen and Warren^2,3^, and Gerin-Lajoie and Warren^4^ on path selection and obstacle avoidance. Baxter and Warren investigated whether the differences in relative distance, or the deviation angle of the ends of the obstacle would regulate path selection. Participants in Baxter and Warren’s study walked at a normal pace in a virtual environment toward a goal (a pole) while avoiding a rectangular shaped obstacle located between the start position and the goal. After walking 1.5 m, the obstacle appeared with the goal visible behind it. As the participant approached, the goal and barrier disappeared, and the next trial began. Obstacle orientation and lateral position were manipulated to obtain differences in relative distance and differences in deviation angle of the ends of the obstacle. The analysis of relative distance and deviation angle measures showed that during obstacle avoidance participants chose a pathway that minimized relative distance and deviation angle. Moreover, the effects of relative distance and deviation angle were independent and additive. Baxter and Warren concluded there are two alternative options for path selection when an obstacle exists in the pathway to an intended endpoint. First, participants might select the path that has a smaller deviation angle from the straight line. Second, participants might select the path that is closer to the edge of the obstacle. In addition, Baxter and Warren expressed the view that rather than planning and evaluating alternative paths to the final goal, the path is emergent, and depends on the evolution of relative distance and deviation angle during walking.

The present study paralleled the Baxter and Warren study with some noteworthy differences. The participant walked at a self-selected pace in a real environment (as opposed to a virtual environment) along a walkway to a target (cup) located on a bookcase shelf, and encountered an obstacle along the way. The target and obstacle were visible at the beginning of a trial in contrast to not appearing until after the participant walked some distance. Thus, we were somewhat confident that path planning was performed at the beginning of a trial. The participant was required to act on the target by picking it up when they arrived at the bookcase. This requirement is different from having the barrier and goal disappear before the participant’s arrival. The most important difference between this study and that of Baxter and Warren is that we included an avoidance margin that was specified as the distance between the outer edge of the obstacle and the edge of the walkway. Thus, the avoidance margin was the available space through which the participant could walk to circumvent the obstacle without encountering the obstacle or crossing the walkway boundary. Other studies have demonstrated the importance of an avoidance margin which has been defined as the distance between the participant’s body and the edge of the obstacle during obstacle avoidance. These studies have demonstrated that participants will maintain an avoidance margin between themselves and the obstacle, and that this distance depends on the characteristics of the obstacle^5–16^. Previous path planning models have described the effects of deviation angle and/or minimum distance from an obstacle on path planning^1,2^. However, we considered avoidance margin, as well as deviation angle and minimum distance from the obstacle as factors related to path planning. Finally, we incorporated measurement of visual gaze into our study.

Although path planning may be achieved using different sensory systems such as audition^17^ to inform the motor system of adjustments necessary for path selection, the visual system provides the most precise environmental information about the pathway^18,19^. Obstacle avoidance models have not properly incorporated the use of gaze and vision to guide actions and avoid obstacles. For example, the recent Baxter and Warren model is based on the Fajen and Warren^3^ model, and neither of these models describe how gaze selection affects decision making regarding obstacle avoidance. A number of obstacle avoidance studies have examined the importance of gaze selection during obstacle avoidance, and they have shown that individuals need appropriate information about the target and obstacle location^20–23^. Gaze patterns have been found to indicate how the performer selects the information to be encoded^24–26^. Previous studies on goal directed gait demonstrated that participants adjust their gaze characteristics such as position and duration^19,27,28^ during performance. The order in which gazes are made during path planning may provide critical information not only about what environmental information is being extracted and used as a person walks to a target, but also about the order in which that information is obtained. Several studies have addressed gaze sequence during daily activities^18^, reaching^24,29^, precision-walking^18,28–32^, and obstacle avoidance^21^. Even when previous studies have made it clear that gaze selection plays an important role in obstacle avoidance, they have failed to relate avoidance margin as an issue of uncertainty. It has been demonstrated that there is a relationship between uncertainty and gaze behavior^23,30^. Decreased avoidance margin size may increase uncertainty in path planning. By including gaze as a factor in path selection, we can gain a better understanding of decision-making while avoiding an obstacle.

The unique contribution of this study was determination of how avoidance margin and target location interact along with gaze selection behavior (gaze allocation and gaze sequence) in path planning when a person walks to a target located on a bookcase shelf. The description of path planning by Baxter and Warren is dichotomous by which individuals may use either the deviation angle from the straight line or the edge of the obstacle to plan a path. In contrast, we provide a multivariate model for weighting of different sources of information used by participants during path planning. To gain a better understanding of the role played by target location on path selection we also subjectively assessed participants’ path planning for different target locations. This test allowed us to determine a threshold value for target location along the bookcase shelf at which the path direction around an obstacle would be changed.

## Methods

### Participants

Twelve normally sighted college students (mean age ± standard deviation: 20.3 ± 1.4 years, 10 female) participated in the study. All participants completed the Edinburgh Handedness Inventory^33^, and were right-hand (score 86.1 ± 9.05) and right-foot dominant. None of the participants had a history of neurological and musculoskeletal disorders, or any other conditions which limited their mobility at the time of participation, according to self-report. The experimental protocol was approved by, and performed in accordance with, the relevant guidelines and regulations of the Indiana University Bloomington Institutional Review Board and conformed to the standards set by the Declaration of Helsinki. All participants provided written informed consent prior to participation.

### Apparatus

**Figure 1:**
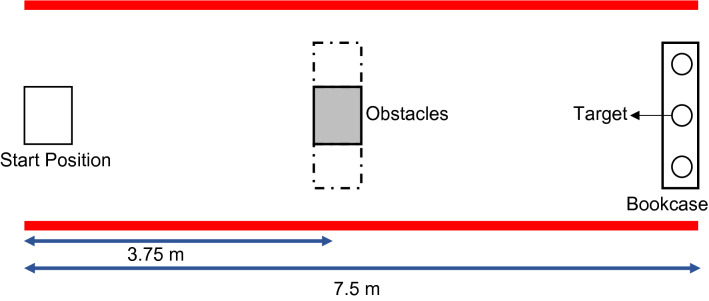
Experimental setup. Participants walked 7.5 m to a bookcase that had an empty 16 oz cup (the “target”) sitting on its lower shelf (.88 m from the floor).

Figure 1 shows the experimental set-up. The start position was located 7.5 m away from and directly opposite to a 1.84 m (height) × 0.90 m (width) × 0.22 m (depth) bookcase with two shelves. The lower shelf was 0.88 m above the ground. An empty 16 oz cup (the “target”) measuring 15.87 cm in height and 8.30 cm in diameter and weighing 275.23 g was on the lower shelf. Three wooden boxes each measuring 45 cm (length) × 32 cm (width) × 24 cm (height) were used to create three obstacles of different widths by arranging the boxes end-to-end so that the total width of the obstacles were 45 cm (one box), 90 cm (two boxes), and 135 cm (three boxes). These obstacles were arranged across the middle of the walkway which was 213 cm in width. The left and right edges of the walkway were marked by a 5 cm wide strip of red tape. The right avoidance margin (AM-R) was the distance between the right outer edge of the obstacle and the right edge of the walkway, and the left avoidance margin (AM-L) was the distance between the left outer edge of the obstacle and the left edge of the walkway.

Participants’ eye movements were measured using a mobile eye tracker from Pupil Labs. Eye movement data was updated at 120 Hz. The eye-tracking software developed by Pupil Labs^34^ was used to extract eye tracking data from the recorded videos. A nine-point calibration was performed.

Participants’ position in the room was tracked using two Microsoft Kinect cameras. A customized visual studio C++ application based on the Kinect SDK 2.0 was developed and used to detect, track, and record the human motion for post-analysis. For more details of the eye tracker and Kinect setup, see Saeedpour-Parizi et al.^35^.

### Task and design

The task was to walk from the start position to the bookcase at a self-selected pace and pick up the target positioned on the lower shelf while avoiding an obstacle and not stepping outside the walkway boundary. There were eight obstacle conditions (see Table 1). Each obstacle condition was determined by left-side and right-side avoidance margins. In Condition 1 (NOBST), no obstacles were present within the walkway. This condition provided a baseline for validation of the assumption in the literature^1^ that the path taken to the target from the start position would be a straight-line in the absence of an intervening obstacle. This condition also provided a baseline from which the findings for the other obstacle conditions could be interpreted.

**Table 1:** Obstacle conditions. AM-L represents the left avoidance margin; AM-R represents the right avoidance margin.

Condition	Obstacle length	Obstacle location	AM-L (cm)	AM-R (cm)
1	No obstacle	NA	NA	NA
2	45 cm	Center of the walkway	83.5	83.5
3	90 cm	Center of the walkway	61.0	61.0
4	90 cm	Right side of the center of walkway	84.0	40.0
5	90 cm	Left side of the center of walkway	40.0	84.0
6	135 cm	Center of the walkway	39.0	39.0
7	135 cm	Right side of the center of walkway	52.0	25.0
8	135 cm	Left side of the center of walkway	25.0	52.0

The avoidance margin conditions were crossed with three target location conditions. The target was positioned either on the center of the lower bookcase shelf, 30 cm to the left of the lower bookcase shelf center, or 30 cm to the right of the lower bookcase shelf center. Participants walked to the target three times for each of the avoidance margin conditions. These experimental manipulations resulted in an 8 (avoidance margins) × 3 (target position) × 3 (trials) repeated measures design.

### Procedure

At the beginning of each trial, participants stood with their eyes closed and barefoot at the start position with both feet side-by-side, and shoulder width apart. Participants were advised that they would receive a verbal “ready” command followed by a verbal “start” command from the experimenter. They were instructed to open their eyes and commence walking to the bookcase and pick-up the target only after hearing the “start” command. Participants were instructed to walk at a comfortable, self-selected pace to the bookcase and pick-up the target. On trials for which an obstacle was present, participants were instructed to avoid making contact with the obstacle, but that they were free to take any path they liked to reach the bookcase and pick-up the target. Therefore, participants were not instructed about which side of the walkway they should use to walk to the target. Participants were also instructed not to step on or over the red tape marking the boundary of the experimental walkway. After participants had picked up the target at the end of a trial, they placed the target back to its original position and returned to the start position.

Upon completion of performing the eight obstacle conditions for each of the target positions, participants were administered a path selection judgement test. For this test, participants were asked to stand at the start position and the target was placed in the center of the lower shelf of the bookcase. The walkway setup was the same as for Condition 3 (i.e., an obstacle was placed in the middle of the walkway such that the left and right avoidance margins were 61 cm each). The target position was then successively changed by 5 cm to the left side up to a distance of 35 cm from the center of the shelf. Participants were asked to rate on a 0–10 Likert Scale which side of the obstacle they would walk around to reach the target when it was in the middle of the shelf and for each 5 cm displacement of the target. The same procedure was repeated by moving the target from the center to the right side of the bookcase shelf.

### Data pre-processing

#### Path data

**Figure 2:**
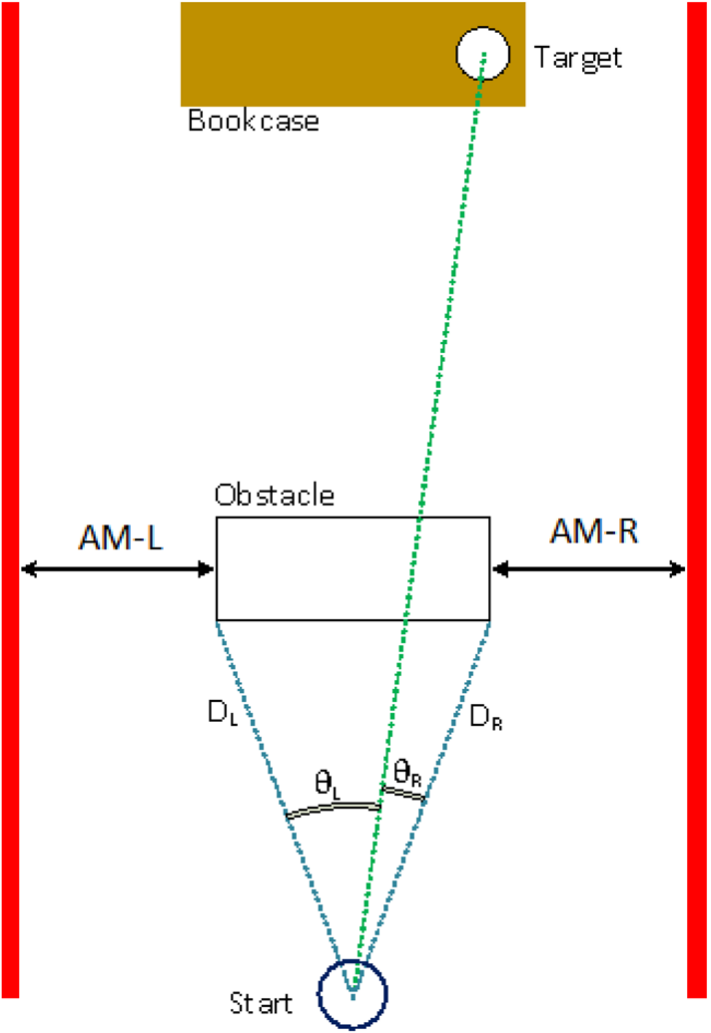
Experimental setup. Participants walked to a bookcase that had an empty 16 oz cup (the “target”) sitting in its lower shelf (.88 m from the floor). AM-L represents the left avoidance margin; AM-R represents the right avoidance margin; D_L_ and D_R_ correspond to the distance from the center of the starting position to the left and right edges of the obstacle, respectively. θ_L_ is the left deviation angle and is the angle between the straight line connecting the target and the center of the starting position and the line connecting the outer left edge of the obstacle relative to the starting position. θ_R_ represents the right deviation angle and is defined as the angle between the straight line connecting the target from the center of the starting position and the line connecting the outer right edge of the obstacle relative to the center of the starting position.

In this study there were three parameters that may have affected obstacle avoidance: a.) relative distance to the obstacle; b.) relative angle to the target; and c.) avoidance margin. As shown in Fig. 2, D_L_ and D_R_ correspond to the distance from the center of the starting position to the left and right edges of the obstacle, respectively. Relative distance is the difference in the distance between the right and left sides of the obstacle and can be computed as ΔD = D_R_ − D_L_. If ΔD was equal to zero, the distance to the right and left sides of the obstacle were equal. If ΔD was positive, the distance to the right side of the obstacle was longer than the distance to left side of the obstacle. The converse is true for a negative ΔD*.*

The angle between the straight line connecting the target from the starting position and the line connecting the outer left edge of the obstacle from the starting position is the left deviation angle (θ_L_, see Fig. 2). Similarly, θ_R_ represents the right deviation angle and is defined as the angle between the straight line connecting the target from the starting position and the line connecting the outer right edge of the obstacle from the starting position. The relative angle is the difference in the deviation angle between the right and left sides and can be calculated as Δθ = θ_R_ − θ_L_. Positive Δθ indicates that the right deviation angle was larger than the left deviation angle. The converse is true for negative Δθ. When Δθ = 0, the deviation angles of the right and left sides were equal. The deviation angle from the straight line in the NOBST condition was also calculated for different target positions.

As previously described, AM-R represents the right avoidance margin and was calculated as the distance between the outer edge of the right obstacle and the right edge of the walkway. Similarly, AM-L represents the left avoidance margin and was calculated as the distance between the outer edge of the left obstacle and the left edge of the walkway. Avoidance margin ratio (AMR) was calculated as AM-R divided by AM-L. An avoidance margin ratio equal to 1.0 means that the right and left avoidance margins were equal in size. An avoidance margin ratio greater than 1.0 means that the right avoidance margin was greater than the left avoidance margin. The converse is true for avoidance margins less than 1.0.

Variations in the difference in relative distance (ΔD), relative angle (Δθ) and the avoidance margins (AM-R and AM-L) were achieved by manipulating the obstacle orientation and target positions. For example, when obstacles were positioned in the middle of the pathway, ΔD was equal to zero and the AM-R and AM-L were equal in value. In this situation, if the target was located in the middle of the shelf, Δθ was also zero. When the target was moved to the left or right, Δθ was positive or negative in value, respectively. When obstacles were moved to the right side of the pathway, ΔD was positive, and AM-R was smaller than AM-L. When obstacles were moved to the left side of the pathway, ΔD was negative, and AM-R was greater than AM-L. For each participant, we calculated Δθ, ΔD, AM-R, and AM-L for a total of 24 levels (8 levels of obstacle × 3 levels of target position)*.*

#### Gait speed

For each participant, gait speed was calculated for each trial of the obstacle and target conditions. As described by Dolatabadi et al.^36^, gait speed was considered as the displacement of the ankle along the anterior–posterior (AP) plane divided by the elapsed time between the start of the first and completion of the last swing phase. Two measures of gait speed were calculated. These were the average gait speed for the entire pathway, and the average gait speed to the middle of the avoidance margin.

#### Start foot analysis

Based on the data collected by the Kinect cameras, the number of trials for starting with the right and left foot was determined for each obstacle and target position.

### Eye tracking data

#### Calculating gaze allocation

Gaze behavior across the different target and obstacle conditions was analyzed to determine the information used by participants for path selection. Records of each participant’s trials were processed manually. To calculate gaze allocation, for each frame of the eye recordings, we found the 2D gaze vector and projected that vector to the ground plane with the reference set at the start position. As a result, gaze allocation in each trial was calculated using a Gaussian density estimate. These Gaussians were normalized by the total duration of each trial. When we found the coordination of gaze allocation for each trial, we scaled the result from 0 to 10 and drew a 3D counter plot for each of the different avoidance margins. The total number of recorded frames in each trial was larger than the number of frames with gazes on the three possible locations (target, avoidance margin, and path area) because not all of the participants’ gaze vectors intersected the three possible locations. Gaze frequency was also calculated as the sum of gazes on each of the areas of interest for trials.

#### The gaze object sequence

**Figure 3:**
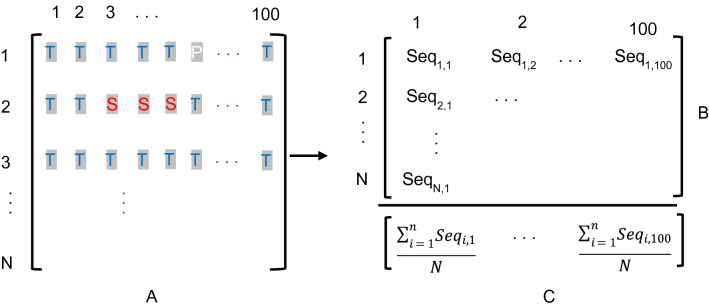
Gaze object sequence calculation procedure. In matrix A, “T” indicates gazes on the target area, “P” indicates gazes on the path area, and “S” indicates gazes on the avoidance margin area. The colors in the figure correspond to the color-coded objects (Blue: Target, White: Path, and Red: Avoidance Margin). Each raw gaze object sequence was represented by a (1 × 100) set of frames. In the first line of this example, the gaze object transitioned from the target to path and then to the target. “N” indicates number of trials in each avoidance margin condition. Matrix B shows a convert matrix. We scaled the result to integer numbers (e.g., “2” indicates target area, “5” indicates path, and “10” indicates avoidance margin). Matrix C shows average result.

To assess gaze order, we quantified each participant’s gaze object sequence and the duration of their gaze sequence. The models of Haji Fathaliyan et al.^37^ and Pan et al.^38^ were used to calculate gaze object sequence. Gazes on the area of interests were analyzed. A color was assigned for each area of interest. These were red for avoidance margin, blue for target, and white for path. Gazes outside of these areas of interest were not analyzed. Because different trials had different durations, each trial was normalized to the total duration and divided into 100 equal segments (see Fig. 3). Figure 3 presents an example of the gaze sequence analysis derived by converting gazes to matrix form. This resulted in eight matrices, and each matrix represented one of the eight avoidance margin conditions. For each avoidance margin condition there were three matrices. These were Matrices A, B, and C. In Matrices A and B different rows (N) indicate the number of trials in each avoidance margin condition, and each column represents the gaze position. In matrix A, “T” indicates gazes on the target area, “P” indicates gazes on the path area, and “S” indicates gazes on the avoidance margin area. In the first row of Matrix A of this example, the gaze position transitioned from the target to path and then to the target. For Matrix B, we scaled the data of matrix A to integer numbers which were arbitrarily determined. The number “2” indicated the target area, the number “5” indicated the path area, and the number “10” indicated the avoidance margin. Matrix C shows the average of the numbers (of 2, 5, and 10) for each of the 100 trial segments. Based on Matrix C, a plot for every avoidance margin condition was generated.

To compare gaze sequences in different obstacle positions we used the “ScanMatch” model^39^ which is based on the Needleman-Wunsch algorithm used in bioinformatics to compare DNA sequences. This model aligns one string with one another to maximize a similarity score. For this reason, we calculated a similarity score between different trials. The means of the similarity scores between obstacle positions were calculated.

#### Avoidance margin gaze ratio

To assess the effect of gazes on avoidance margins for path selection, we quantified cases for each participant on the left and right avoidance margins. Then, we defined avoidance margin gaze ratio (AMGR) as described in Eq. (1):

1 $$AMGR = \frac{{\left( {Gaze \;time \;on \;Right\;AM - Gaze\;time\;on\;Left\;AM} \right)}}{{\left( {Gaze\;time\;on\;Right\;AM + Gaze\;time\;on \;Left \;AM} \right)}}$$

AMGR is a value between − 1 and + 1. An AMGR equal to zero means that gazes on the right and left avoidance margins were equal in time. An AMGR greater than zero means that the gazes on the right avoidance margin were longer than the gazes on left avoidance margin. The converse is true for AMGR less than Zero.

### Data analysis

Multiple logistic regressions were performed on ΔD and AMR when accounting for Δθ (when the target was in the middle, left, and right positions on the bookcase shelf) to assess the relationship of each of these parameters on path planning. A univariate logistics regression was performed on Δθ to assess the relationship of this parameter on path planning. Ground truth binary labels were Right side = 1 and Left side = 0.

To assess the effect of all parameters together (ΔD*,* Δθ, AMR) on path selection, a multivariate analysis model was performed. This model also accounted for the interaction of ΔD and AMR with Δθ. To assess the effect of these parameter weights, we used a bootstrapping technique with random shuffling of 2000 surrogate data. We then used a machine learning logistic regression model with a sparse set of features determined by L_1_ regularization (LASSO) to predict whether a selected path was to the right or left. To evaluate model performance, we used a leave-one-out cross validation method^40^. We then calculated the “Variance account for each of the parameters” in path planning. The Logistics regression was implemented in Python using the scikit-Iearn machine-learning library. To assess the effect of different parameters at an individual level, a multilevel analysis^41^ was performed to find the weight that each participant assigned to each of the decision variables.

An 8 (obstacle condition) × 3 (target position) repeated-measures ANOVA was conducted on average gait speed to assess how speed changed as a result of the different obstacle conditions and target positions.

An 8 (obstacle condition) × 3 (target position) × 3 (gaze position) repeated-measures ANOVA was conducted on gaze frequency to assess how gaze behavior changed as a result of the different obstacle conditions and target positions. Distribution of gaze allocation was also assessed by the HyBayes package^42^.

A Kolmogorov–Smirnov test was used for different avoidance margin conditions to assess the normality assumption of similarity scores for gaze sequence. The PROPER method proposed by Jahandideh et al.^43^ was used for comparison of similarity scores. A one-way ANOVA was conducted on similarity scores between Conditions 2 to 8 and the NOBST condition to assess how gaze sequence behavior changed as a result of different avoidance margins. Bonferroni corrected post hoc comparisons were used to further investigate the effect of the avoidance margin condition on the gaze sequence.

Univariate logistic regressions were performed to assess the effect of AMGR on path planning. Ground truth binary labels were Right side = 1 and Left side = 0. To assess the effects of AMGR and AMR on path selection, a multivariate analysis model was performed.

An independent analysis for trials was not significant and the trial scores were therefore averaged. All analyses were performed in Python, and Greenhouse–Geisser epsilon was used to control for violations of sphericity. An alpha level of .05 was used for all tests.

### Ethical approval

All procedures were approved by the Indiana University Bloomington at Institutional Review Board.

## Results

### Deviation angle from straight line in the NOBST condition

When the target was in the middle of the bookcase shelf in the NOBST condition, participants walked straight to the target. However, when the target was located either to the right or left side of the bookcase shelf participants did not walk straight to the target to pick it up. Instead, when the target was located to the right-side, the deviation angle from the straight line was 3.05 ± 0.15 degrees (Mean ± SD). The converse was true when the target was positioned to the left since the left side deviation angle was 2.61 ± 0.20 degrees (Mean ± SD) from the straight line to the target.

### Path planning results

**Figure 4:**
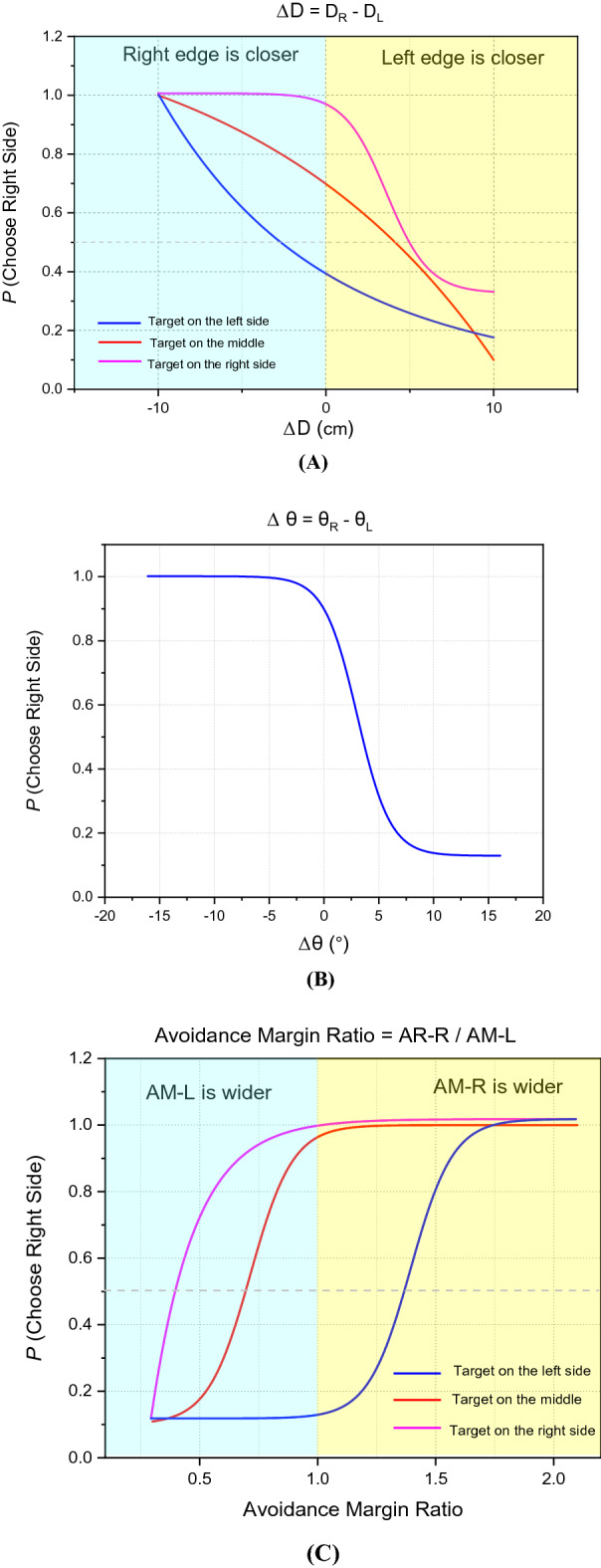
Probability of walking to the right side of the obstacle based on the target and obstacle locations. (**A**) Influence of ΔD on the probability of walking to the right of the obstacle. (**B**) Influence of Δθ on the probability of walking to the right of the obstacle. (**C**) Influence of avoidance margin on the probability of walking to the right of the obstacle.

The probability of walking to the right of the obstacle for different decision-making parameters is shown in Fig. 4. Figure 4A shows the influence of ΔD on the probability of walking to the right of the obstacle for different target locations. It can be seen when ΔD was equal to zero (when the obstacle was equidistant from the starting position), the probability of walking to the right side was 70% when the target was located in the middle, was 95% when the target was located on the right side, and was 40% when the target was located on the left side of the bookcase. As ΔD became negative (the distance to the right edge of the obstacle was less than the distance to the left edge of the obstacle), the probability of walking to the right-side increased for all the target locations. When ΔD was equal to, or less than, − 10 cm, participants always walked to the right side of the obstacle (the probability was equal to 100%). Also, when ΔD was positive (when the distance to the left side of the obstacle was less than the distance to the right side of the obstacle), the probability of walking to the right side of the obstacle decreased. When ΔD was greater than 5 cm (the left edge of the obstacle was 5 cm closer than the right edge of the obstacle), the probability of walking to the left side of the obstacle was equal to, or greater, than walking to the right side.

Figure 4B shows the influence of Δθ on the probability of walking to the right of the obstacle. It can be seen that when Δθ was equal to zero (the target was positioned on the middle of the shelf), the probability of walking to the right side of the obstacle was 90%. As Δθ became negative (the target was positioned on the right side of the shelf), the probability of walking to the right-side increased. Notably, when Δθ was equal, or less than − 5°, participants always walked to the right side of the obstacle (the probability was equal to 100%). Also, for positive Δθ measures (the target was positioned on the left side of the shelf), the probability of walking to right side decreased.

Figure 4C shows the influence of the avoidance margin on the probability of walking to the right of the obstacle. It can be seen that when the target was in the middle of the bookcase shelf (red line), and the avoidance margin was equal to 1 (the situation where the right and left avoidance margins were equidistant from the edge of the pathway), the probability of walking to the right side was approximately 90%. With an increase of the width of the right avoidance margin relative to the width of the left avoidance margin, the probability of walking to the right side of the obstacle increased. However, when the width of the left avoidance margin increased, participants started walking to the left side of the obstacle. When the target was on the right side of the bookcase shelf (pink line), and the avoidance margin ratio was 0.5, participants walked to the right side of the obstacle. When the target was on the left side (blue line), and the avoidance margin ratio was 1.4, participants walked to the left side of the obstacle.

### Logistics regression analysis

Table 2 shows the result of the univariate logistics regression analysis. It can be seen that when the target was located on the left side of the bookcase shelf (increasing Δθ), the logit-probability of walking to the right side significantly decreased by a coefficient of 0.34. Also, when the target was in the middle of the bookcase shelf and ΔD increased (the distance to the right edge of the obstacle increased as opposed to the distance to the left edge) the logit-probability of walking to the right side significantly decreased by a coefficient of 0.35. The AMR coefficient shows that when the ratio of the width of the right avoidance margin and left avoidance margin increased by 1, and the target was in the middle of the bookcase shelf, the logit-probability of walking to the right side significantly increased by a coefficient of 4.67. When the target was on the left side of the bookcase shelf, and ΔD increased, the probability of walking to the right side significantly decreased by coefficient of 0.65. Furthermore, increasing AMR resulted in a significant increase in the probability of walking to the right side of the obstacle by a coefficient of 5.13. However, when the target was on the right side of the bookcase shelf, participants walked to the right side of the obstacle without consideration of ΔD and AMR. The individual level analysis is presented in Supplementary Figs. 2–4 and Supplementary Table 1.

**Table 2:** Regression coefficient and *p*-values for univariate regression analysis.

Feature	Regression coefficient	*p* value
Δθ	− 0.34	< .01
**Target on the middle**		
ΔD	− 0.35	< .01
AMR	4.67	< .01
**Target on the left side**		
ΔD	− 0.65	< .01
AMR	5.13	< .01
**Target on the right side**		
ΔD	0.55	.87
AMR	43.17	.99

### Multivariate logistics regression analysis

Table 3 shows the confidence intervals for the multivariate logistics regression. It can be seen that all parameters and interactions contributed to the model (*p* < .05). All three predictor variables played a significant role in the decision about the path to take to the target (*p* < .05). The model accuracy using LOOCV reached 85.1 ± 2.07% (mean ± SD). It can be seen in Table 3 that the most important factor which accounted for more than 37% of the variance in path planning was Δθ (an indicator of target position) and ΔD accounted for more than 36% of the variance in path planning. However, including AMR in the model accounted for approximately 26% of the variance in path planning decision making.

**Table 3:** Estimated effect, 95% confidence interval (CI), and ratio to total effect on path planning in the multivariate analysis with bootstrapping technique with random shuffling of 2000 surrogate data.

Feature	Estimated effect	CI 95%	*p* value	Ratio to total effect on path planning (%)
Δθ	− 0.17	(− 0.20, − 0.14)	< .01	37.69
ΔD	− 0.06	(− 0.09, − 0.03)	< .01	36.07
AMR	− 0.22	(− 0.42, − 0.01)	.036	26.22
Δθ: ΔD	0.01	(0.10, 0.15)	< .01	
Δθ: AMR	0.12	(0.00, 0.01)	< .01	

### Speed analysis

**Figure 5:**
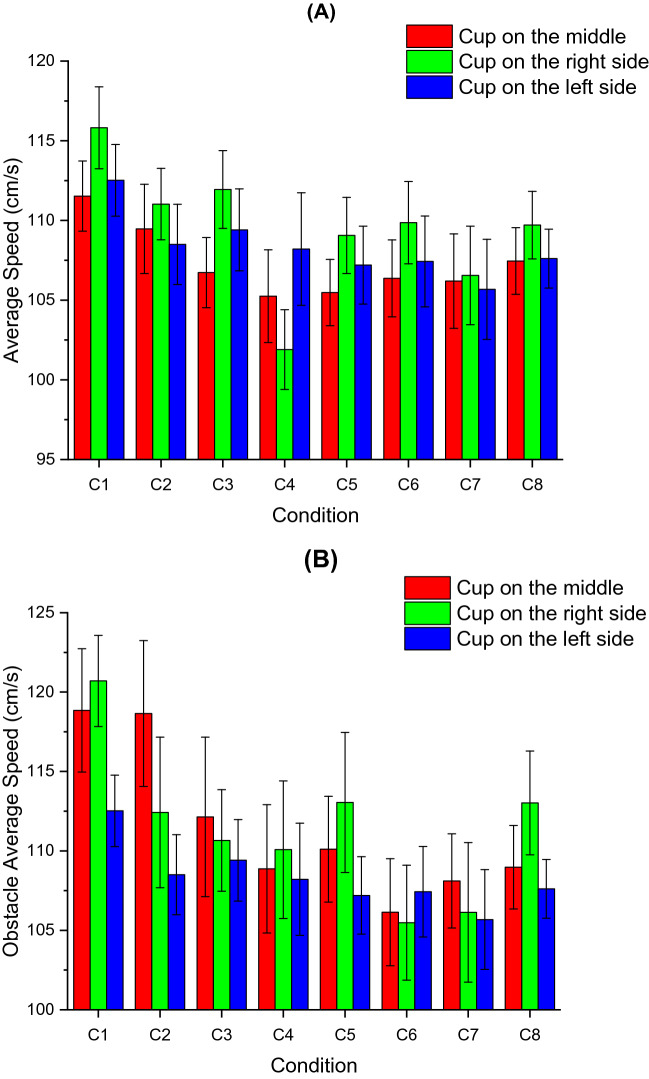
Average speed as a function of target location and obstacle condition. (**A**) Average velocity from the start location to bookcase (**B**) Average speed from the start location to the obstacle area. The obstacle conditions are C1: Condition 1 (NOBST), C2: Condition 2 (AM-R80/AM-L80), C3: Condition 3 (AM-L61/R61), C4: Condition 4 (AM-L84/R40), C5: Condition 5 (AM-L40/R84), C6: Condition 6 (AM-L39/R39), C7: Condition 7 (AM-L52/R25), C8: Condition 8 (AM-L25/R52). Error bars indicate SE.

Figure 5 shows the average gait speed for different target locations under different conditions. Figure 5A shows the average speed between the start and target locations. Figure 5B shows the average velocity between the start and obstacle area. It is notable that the NOBST condition was faster than the other conditions. In general, the average velocity decreased as the avoidance margin decreased. In Condition 6, the average obstacle speed declined dramatically. The avoidance margins for both sides in this condition is the smallest of all conditions. As a result, the participant had to slow down to successfully cross the obstacle.

The ANOVA performed on averaged speed showed there were significant main effects for obstacle condition, *F*(7, 77) = 3.11, *p* < .01, and target location, *F*(2, 22) = 4.81, *p* = .018. In addition, the Obstacle × Target Location, *F*(14, 154) = 2.44, *p* < .01 interaction was significant. The ANOVA performed on the obstacle averaged speed showed there was a significant main effect for obstacle condition, *F*(7, 77) = 7.68, *p* < .01. However, the target location and interaction of Obstacle × Target Location was not significant.

### Start foot analysis

The Friedman test indicated that starting with the right foot or the left foot was significant for different obstacle and target positions (*χ*^2^ = 15.97, *p* = .001). The frequency of starting with the right foot when the target was on the middle or on the right side of the shelf was in the range of 65% to 66%, as shown in Table 4. Starting with the right foot decreased to 52% when the target was placed on the left side of the bookcase shelf. The Pearson correlation coefficient between the starting foot and selected path for different obstacle and target position was 0.53 (*p* = .03).

**Table 4:** Frequency (%) of choosing the right foot as the starting foot cross the 8 obstacle conditions. Obstacle conditions are as 1: Condition 1 (NOBST), 2: Condition 2 (AM-R80/AM-L80), 3: Condition 3 (AM-L61/R61), 4: Condition 4 (AM-L84/R40), 5: Condition 5 (AM-L40/R84), 6: Condition 6 (AM-L39/R39), 7: Condition 7 (AM-L52/R25), 8: Condition 8 (AM-L25/R52).

Cup position	Condition 1	Condition 2	Condition 3	Condition 4	Condition 5	Condition 6	Condition 7	Condition 8
	Choose right foot as the start foot (%)							
Middle	55%	90%	85%	45%	55%	80%	70%	40%
Left	60%	60%	45%	30%	60%	60%	55%	45%
Right	55%	80%	75%	55%	60%	65%	45%	60%

### Eye tracking results

#### Gaze allocation

**Figure 6:**
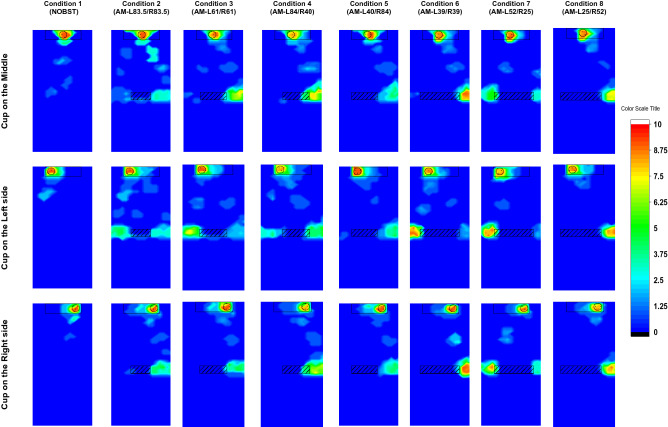
The gaze allocation on the target location, path area, and avoidance margins (hatched lines indicate obstacle and solid circle indicates cup position).

Figure 6 shows the gaze allocation analysis for the different obstacle conditions and target positions. It can be seen that when avoidance margin decreased, gaze allocation in the avoidance margin area increased. The repeated measures ANOVA performed on the gaze frequency showed that there were significant main effects for obstacle condition, *F*(7, 77) = 3.446, *p* < .01, and gaze position, *F*(2, 22) = 806.64, *p* < .01. In addition, Obstacle Condition × Gaze Position, *F*(14, 154) = 39.383, *p* < .01, Gaze Position × Target Position, *F*(4, 44) = 5.586, *p* < .01, and Obstacle Condition × Target Position × Gaze Position, *F*(28, 308) = 11.92, *p* < .01, interactions were all significant.

#### Gaze object sequence

**Figure 7:**
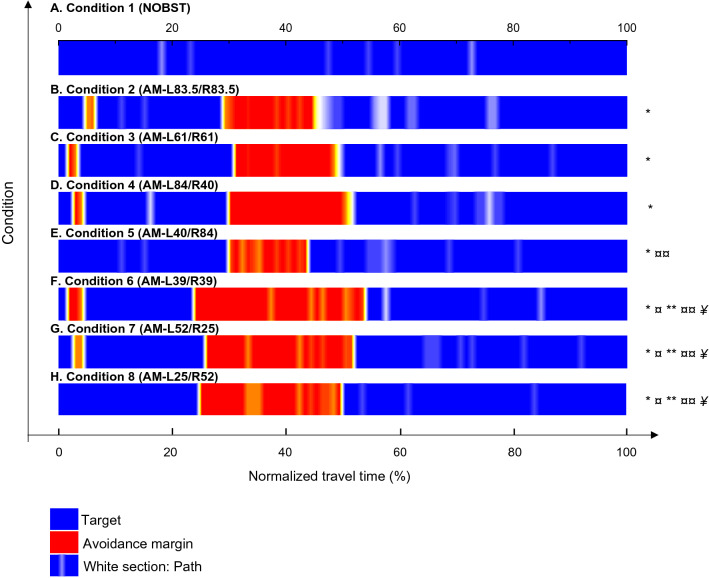
Characteristic gaze object sequences were produced using dynamic time warping barycenter averaging over gaze data from participants for each of the 8 obstacle conditions (**A**–**H**). The colors in the figure correspond to red as avoidance margin area, white as path area, and blue as target area. The lengths of the sequences were normalized to 100 segments for visualization. *p < .05 as opposed to Condition 1, ¤*p* < .05 as oppose to Condition 2, **p < .05 as oppose to Condition 3, ¤¤*p* < .05 as oppose to Condition 4, and ¥*p* < .05 as oppose to Condition 5.

Figure 7 is a visual representation of the gaze object sequence for each of the eight obstacle conditions. Blue sections indicate gaze on the target area, Red sections indicate gaze on the avoidance margin area, and the White sections indicate gaze directed within the path area. Some differences in gaze sequence were observed within the initial 60% of the trial time. These differences included variable amounts of time spent looking at the target area and obstacle. However, during the last 40% of the total trial time, no differences were observed between the different obstacle conditions.

Table 5 shows the similarity scores from the different gaze object sequences computed across the different obstacle conditions. Kolmogorov–Smirnov tests showed that the distributions of the similarity scores across the different obstacle conditions were normal (*p* > .05). There was a greater similarity score within obstacle conditions as shown with the dashed squares. This indicates that participants had similar gaze sequence behavior within each obstacle condition. Similarity scores between the NOBST condition and those conditions with an obstacle were small as supported by a significant effect for condition, *F*(6,9065) = 11,014, *p* < .01, indicating that gaze sequence changed as a function of avoidance margin. Post hoc comparisons showed that Condition 2 significantly differed from Conditions 6, 7 and 8 (*p* < .05). Condition 3 significantly differed from Conditions 6, 7 and 8 (*p* < .05). Condition 4 significantly differed from Conditions 5, 6, 7 and 8 (*p* < .05). Condition 5 significantly differed from Conditions 6, 7 and 8 (*p* < .05). All other remaining comparisons were not significant.

**Table 5:**
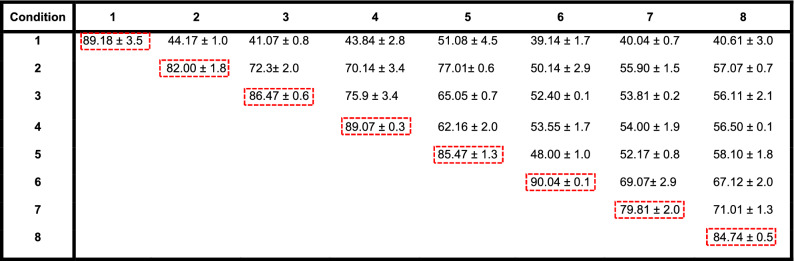
Mean (± SD) similarity scores of gaze sequences across the 8 obstacle conditions. Red dashed squares indicate similarity within each obstacle condition. The numbers 1–8 represent the obstacle conditions as 1: Condition 1 (NOBST), 2: Condition 2 (AM-L80/R80), 3: Condition 3 (AM-L61/R61), 4: Condition 4 (AM-L84/R40), 5: Condition 5 (AM-L40/R84), 6: Condition 6 (AM-L39/R39), 7: Condition 7 (AM-L52/R25), 8: Condition 8 (AM-L25/R52).

#### Effect of gazes on avoidance margin on path selection

**Figure 8:**
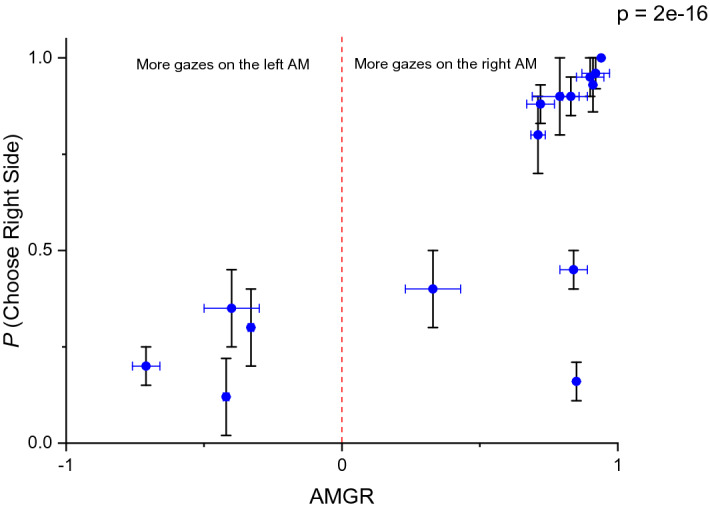
Probability of walking to the right side of the obstacle based on the avoidance margin gaze ratio (AMGR). The dashed line is zero AMGR (gazes on the right and left avoidance margin were equal in time). *p* value indicates significant level of univariate logistics regression. Black error bars represent SE for Y-Axis and Blue error bars represent SE for X-Axis.

The probability of walking to the right of the obstacle as a function of AMGR is shown in Fig. 8. It can be seen that when more gazes were directed on the left avoidance margin (AMGR < 0), the probability of walking to the right side was less than 50% and the participant walked to the left side. However, in most cases, when more gazes were on the right avoidance margin (AMGR > 0), the probability of walking to the right side was more than 50%. It is interesting that in some cases when AMGR > 0 (gazes on the right side were greater than the left side), the participant did not walk to the right side. The relationship between the probability of walking to the right side, AMR, AMGR, and target position is presented in the Supplementary files, Fig. 1.

Table 6 shows the result of the univariate and multivariate logistics regression analysis. It can be seen that when there was a greater number of gazes on the right avoidance margin (AMGR increased), and the logit-probability of walking to the right side significantly increased by coefficient 1.94. Upon multivariate analysis, it was shown that the coefficients of AMR, AMGR, and probability of walking to the right side were positively related.

**Table 6:** Regression coefficient and *p*-values for regression analysis between AMGR and probability of walking to the right side.

Feature	Regression coefficient	*p* value
**Univariate analysis**		
AMGR	1.94	2.00e−16
**Multivariate analysis**		
AMGR	1.41	2.06e−15
AMR	4.57	2.00e−16

#### Target position effect

**Figure 9:**
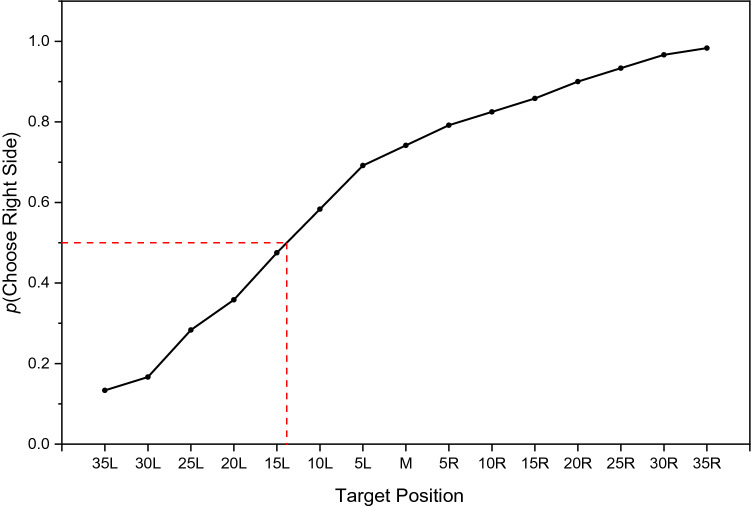
Probability of choosing to the right side of the obstacle as a function of target location when the target was changed by 5 cm successively to the left side or to the right side. M indicates target in the middle, L indicates a shift of the target to the left side and R indicates a shift of the target to the right side. The dashed line shows 50% probability of selecting the right side of the obstacle.

Figure 9 shows the results of the path selection judgement test. The dashed line represents when there was a 50% probability of choosing to walk to the right side of the obstacle/walkway. Probabilities higher than 50% mean that the participant preferred to choose walking on the right side of the obstacle to reach the target. However, it can be seen that participants chose to walk on the left side of the obstacle/walkway when walking to the target when the target was shifted as close as 15 cm to the left side relative to the middle of the shelf.

## Discussion

Path selection around obstacles by participants was systematic. The aim of this study was to discover how people choose their path and the visual information used in path selection. This study examined obstacle avoidance strategies in response to stationary obstacles. The findings of this study provided a multivariate model for the weighting in path selection to a target by combining avoidance margin, deviation angle, and distance to the obstacle. At the outset of a trial the participant assessed the size of the avoidance margin, the deviation angle from the target, and the distance from the obstacle, and weighted the importance of each of these factors, and then made their path selection. This contrasts with Baxter and Warren who provided a dichotomous model for which path selection was limited to either deviation angle or distance to the obstacle. The difference in finding between our studies might reflect Baxter and Warren not considering avoidance margin in their study. Baxter and Warren did not find an interaction effect and concluded that deviation angle and relative distance to the obstacle were additive and independent. In contrast, we found (Table 3) not only main effects but also the interactions of our factors to be significant (deviation angle, relative distance, and avoidance margin) and therefore our model addresses the complexity of decision making for path planning. We also provided an objective measurement of a baseline pathway to the target which has not previously been provided by research on the topic of obstacle avoidance^1,20,44^. Other studies have assumed that participants would take a straight-line path to the target in the absence of an obstacle. We found that the participants took a straight path when the target was on the middle of the bookcase shelf. However, they deviated from the straight-line path when the target was either on the right or left side of the bookcase shelf. We conditioned our data based on the actual deviation angel used by participants. We also included a description of gaze characteristics dependent on the avoidance margin, target, and obstacle relationship to the start position in our study.

The univariate analyses showed deviation angle from the straight line, distance to the left and right edges of the obstacle, and the left and right avoidance margins played key roles in how participants made their path selections (Table 2). Our findings using the deviation angle showed that participants selected a path to the target that had the smallest deviation angle (Fig. 4B). When Δθ was zero and the target was on the middle of the bookcase shelf, there was a 90% probability that the participant would walk to the right side of the obstacle. It has been demonstrated that right-handed individuals tend to use their right hand when performing bimanual tasks^45,46^. This attentional bias might also explain why participants in this study preferred the right path. When Δθ was negative and the target was on the right side of the bookcase shelf, there was a 100% probability that the participant would walk to the right side of the obstacle. Therefore, the participant did not consider the relative distance to the obstacle nor the avoidance margins for path selection. However, when the Δθ was positive (15 deg) and the target was on the left side of the bookcase shelf there was just a 15% probability that the participant would walk to the right side of the obstacle. These findings indicate that participants used a smaller deviation angle which resulted in a less curved path to the target to decide on which path they would take to get to the target. We also found that when the target was on the middle or the left side of the bookcase, the side of the obstacle with the closest proximity to the participant (relative distance) was used for path selection (Fig. 4A). It can be seen in Fig. 4A that when ΔD was equal to zero, there was an 85% probability of walking to the right side of the obstacle. When the right edge of the obstacle was closer to the participant (negative ΔD), all participants chose to walk on the right side of the obstacle as they walked to the target. However, the probability of choosing the right-side decreased when the left edge of the obstacle was closer to the participant. The use of the smallest relative distance for the selected path to the target coincided with the shortest path to the target and reduced the energy usage for path selection to the target. This finding is consistent with Rosenbaum^47^ who showed that participants chose the shortest distance to walk to the target.

Our results also showed that changes in the avoidance margin between the left and right edges of the obstacle affected the path selected to the target (Fig. 4C). Participants chose their path based on the avoidance margin that had the greatest effect of minimizing a collision from occurring. When the right avoidance margin was equal to the left avoidance margin and the target was in the middle of the bookcase, there is an 80% probability of walking to the right side of the obstacle. When the right avoidance margin was wider than the left avoidance margin, participants chose the right side of the obstacle to approach the target. However, the probability of choosing the right-side decreased when the left avoidance margin was wider than the right avoidance margin. In the case when the avoidance margin was greatly reduced in size, but participants had enough space to avoid a collision, they chose the larger avoidance margin to approach the target. It is important to point out here that they chose to not take the shortest path to the target, but instead chose to take the longer path to the target (the one that deviated most from a straight line). This was in spite of the fact they knew they could safely circumvent the obstacle. We suggest this was because they chose the path that required less cognitive effort. The shorter path with a smaller avoidance margin would have required a greater number of adjustments in gait to circumvent the obstacle.

The use of a multivariate logistics regression analysis showed that Δθ, an indicator of target location, was more important than other variables in path selection (Table 3). The second most important variable in this analysis was ΔD which indicates distance to the obstacle. These results jointly indicate that a smaller deviation angle which results in a straighter path to the target is the most important consideration in path selection. This finding indicates that participants preferred the path that minimized local variables D and θ. This finding is consistent with the finding of Gérin-Lajoie and Warren^4^. When the ΔD and Δθ agreed, responses overwhelmingly favored the path with the smallest distance and deviation angle. However, when ΔD and Δθ were in conflict, participants chose the path with the smallest deviation angle (Δθ) to the target and not the path with the smallest distance to the edge of the obstacle (ΔD). A possible reason for this discrepancy is that the straighter path to the target might be more important than shorter or curved paths to the target. Our findings are not consistent with those of Silva et al.^12^ which showed that participants used only the minimum distance for path selection.

We found that the right and left avoidance margins combined accounted for 26% of the variance in decision making related to path planning. Research^11,48^ has demonstrated that individuals tend to initiate an avoidance behavior 4 m from an obstacle. However, to our knowledge there has not been a study which has addressed the importance of avoidance margins in path planning. Our findings are different from those studies that have restricted path selection using vertical poles^8,49^. These studies showed that individuals do not choose the path between two poles when the distance between the poles is less than 1.4 times of their shoulder width. Our results showed that in addition to ΔD and Δθ, our participants may have selected a path with the widest avoidance margin which required the least amount of cognitive effort^50^. Therefore, this finding is consistent with the framework proposed by Harrison et al.^51^ allowing for the influence of “soft” constraints (cognitive effort) together with “hard” constraints (ΔD and Δθ) on behavior. In addition, a study by Nordin et al.^52^ revealed that the activity in supplementary motor area and premotor cortex increased after individuals saw obstacles. Animal studies indicate that the motor cortex is involved in locomotion modifications^53^ such as adjusting gait during obstacle avoidance.

It can be seen in Table 4 that participants preferred using the right foot when the target was on the right side or middle of the bookcase shelf. However, when the target was on the left side of the shelf, there was no preference for a start foot. Our results describing the start foot selection for different obstacle and target positions, therefore, show that normal right foot selection is sometimes violated to accommodate the path selection to the target. These results are consistent with the findings of a recent study^54,55^, which indicated accurate foot placement is controlled by the combination of sensory feedback and an internal feedforward model to accurately estimate the joint movement. Results of a neurophysiology study^56^ indicated that limb selection coordination affected activity in the supplementary motor area.

We found that the participant slowed down when they encountered obstacles compared to the NOBST condition. In our study, we found that a smaller avoidance margin caused a slower average speed toward the obstacle as well as towards the target. The target position also changed the total average speed. Baxter and Warren view their data as reflecting an emergent path dependence on changes in relative distance and deviation angle encountered during walking. This finding may reflect their procedure of not showing the target and obstacle locations to their participants until they started walking. We, however, showed the target and obstacle locations to participants before they started walking. Therefore, participants had time to plan their path to the target, but they used online planning to modulate their gait speed to accommodate the circumvention of the obstacle. As shown in Table 2, when the target was on the right side of the shelf the participants chose the right side without consideration of the avoidance margin. This resulted in faster average speeds in most of the conditions. Because the participants preferred the right side, they used less caution in circumventing this side of the obstacle. Also, the average speed to the obstacle decreased when the avoidance margin was small (Conditions 6, 7, and 8). The results of the study are in line with those of a recent study^5^, which found that individuals slow down their walking speed when they are 2 m from the obstacle. Therefore, our findings indicate that individuals plan ahead to maintain an avoidance margin between themselves and obstacles. Neurophysiology studies^52,56^ indicated that the prefrontal cortex, primary motor cortex, and supplementary motor area are engaged in the planning and programming of a set of motor commands in gait planning.

Knowledge of the intervening obstacle and monitoring body position are necessary for trajectory modifications during gait to the target^21^. The extent to which gaze movement was deployed during visual scene processing in path planning was investigated by comparing gaze distributions and sequences for different obstacle conditions. The allocation of participants' gaze time on the target location, path area, and avoidance margins is shown in Fig. 6. When the obstacle was not present (NOBST condition) the role of vision was modest. In this condition, participants looked primarily at the target location with only a few gazes on the path. Gazes on the target were used to describe the direction of gait to the target location. When an obstacle was present participants looked at the target location, as well as the avoidance margin and path area. A notable finding was that the gaze sequence on the target location, path area, and avoidance margin areas changed with the obstacle condition (Fig. 7). The greatest proportion of gaze time was on the target. As participants got closer to the target (time > 80%), gazes on the path or avoidance margin areas decreased to almost zero. This could be due to participants using their peripheral vision to navigate the path as they approached the target. Earlier along the travel path (time < 10%) there were some gazes on the avoidance margin and target areas. These gazes allowed for path planning and suggest that the use of visual information (Δθ, ΔD, AM-L, and AM-R) was emphasized. Later in the path taken to the target (25% < time < 45%), participants’ gaze shifted to the avoidance margin area. Gazes shifted to the avoidance margins when the width of the avoidance margins was small. After passing the obstacle (45% < time < 80%), there were few gazes on the path area and a greater number of gazes on the target location. Our results collectively support the findings of previous studies^21,44^ showing that visual information is needed for path planning to the target. Participants need to know the location of the target, and this information is provided by gazes on the target and path area. When they encounter an obstacle in the path between them and the target, participants need to know the magnitude of the change in direction necessary to circumvent the obstacle. Gazes on the avoidance margin area are useful for this purpose. While other studies^21,44,57^ have generally showed gaze data is used for safe travel during obstacle avoidance, our analyses specified the magnitude for use of gazes in terms of both allocation and sequencing according to avoidance margins, path, and target area. Results of the gaze sequence similarity scores (Table 5) showed that when there was an obstacle in the path to the target, and there was a decrease in the width of the avoidance margin, a different gaze sequence pattern was used by participants. Figure 8 and Table 6 allow a direct comparison between gaze time on the avoidance margin and path selection. When gaze time was greater on the left avoidance margin (AMGR < 0), the participants chose to walk on the left side of the obstacle as they walked to the target. In most cases when a greater number of gazes were directed toward the right avoidance margin (AMGR > 0), the probability of walking to the right side was greater than 50%. But in some cases when AMGR > 0 was observed (gazes on the right side were higher than on the left side), participants did not walk past the right side of the obstacle as they walked to the target. As a consequence of the increase in the number of gazes toward the right avoidance margin, the participants rejected the option with the smaller avoidance margin by selecting the left side of the obstacle as they walked to the target. Our results suggest that optimum performance requires gaze to be directed in a way that reduces the uncertainty of variables necessary to complete a given task. The present results are consistent with previous findings that link gaze activity to uncertainty reduction in driving^58^ and walking tasks^23,26,30^.

The finding that the target position had the strongest effect on path selection, and that participants chose a path with the smallest deviation angle from the straight path to the target, is of notable importance for understanding obstacle avoidance. The purpose of the path selection judgement test was to provide a more complete analysis of the weighting given to target location in path selection. The findings for the path selection judgment test (Fig. 9) showed that when the target was in the center of the bookcase shelf the probability of walking to the right side of the obstacle was almost 80%. When the target was placed 35 cm on the right from the center of the shelf, the probability of walking to the right side of the obstacle was 100%. When the target was placed 35 cm on the left from the center of the shelf, the probability of walking to the left side of the obstacle was almost 88%. These findings were consistent with our findings in path selection trials for which the participants walked to the target. A notable finding was that when the target was placed 15 cm to the left side of the bookcase center, the probability of walking to the right side and left side of the obstacle was equal. The sensitivity of the path selection decision making process is highlighted by the finding that a change in target location of one additional centimeter to the left side (i.e., to 16 cm), which is the equivalent of 0.076 degrees of change in the visual angle, resulted in the participant changing their path selection to the left side of the obstacle. Therefore, the threshold for participants abandoning their preferred side for circumventing the obstacle was 15 cm to the left of the bookcase shelf center.

## Conclusion

This study investigated how people choose their path and the visual information used for obstacle avoidance. The unique contribution of this study was to provide a multivariate model for the weighting in path selection to a target by combining avoidance margin, deviation angle, and distance to the obstacle. This study showed that participants chose a path with a smaller deviation angle from a straight line to the target. In addition, participants chose a side of the obstacle which was closer to them. We found that the right and left avoidance margins combined to account for 26% of the variability in decision making related to path planning. We found that in some cases participants chose a longer path around the obstacle even when the available avoidance margin which would have resulted in a straight line to the target was large enough to allow passage.

Our gaze analysis findings showed that participants directed their gaze to minimize the uncertainty involved in successful task performance. Gaze allocation was directed to optimize information pick up for successful task performance, and reflected the interaction of target location, path area, and avoidance margins. Gaze sequence changed with obstacle location. Early in participants’ walk to the target, the greatest allocation of gaze was on the avoidance margin and target. Later in participants’ walk to the target, gaze shifted to the avoidance margin when it was small, and then shifted primarily to the target after the obstacle was passed.

Results of the path selection judgment test showed that when the target was placed 15 cm to the left side of the bookcase center, the probability of walking to the right side and left side of the obstacle was equal. Therefore, the threshold for participants abandoning their preferred side for circumventing the obstacle was 15 cm to the left of the bookcase shelf center.

## Supplementary Information

Supplementary Information 1.

Supplementary Information 2.
