# Supplementary material for: Target position and avoidance margin effects on path planning in obstacle avoidance
Source: Sci Rep. 2021 Jul 27;11:15285. doi: 10.1038/s41598-021-94638-y (PMC8316463; doi:10.1038/s41598-021-94638-y)
Supplement: Supplementary file 2 — Supplementary Information 2. [file 41598_2021_94638_MOESM2_ESM.pdf]

Supplementary Information

Title: Target position and avoidance margin effects on path planning in obstacle avoidance

Mohammad R. Saeedpour-Parizi[1,3], Shirin E. Hassan[2], Ariful Azad[3], Kelly J. Baute[4], Tayebeh Baniasadi[1], and John B. Shea[1]

[1] Department of Kinesiology, School of Public Health, Indiana University Bloomington

[2] School of Optometry, Indiana University Bloomington

[3] Department of Intelligent Systems Engineering, Luddy School of Informatics, Computing, and Engineering, Indiana University Bloomington

[4] A Splendid Earth Wellness LLC

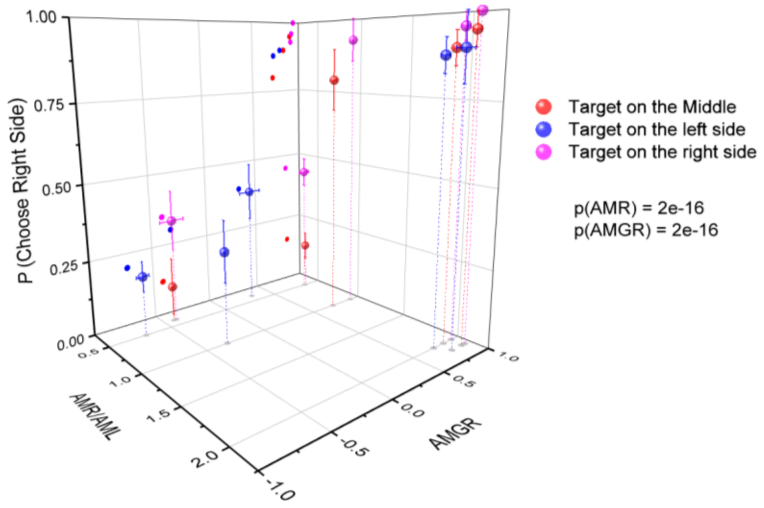

Supplementary Figure 1. Probability of walking to the right side of the obstacle based on the safety margin gaze ratio (AMGR), safety margin ratio (AMR/AML). P value indicates significant level of multivariate logistics regression. Error bars represent SE.

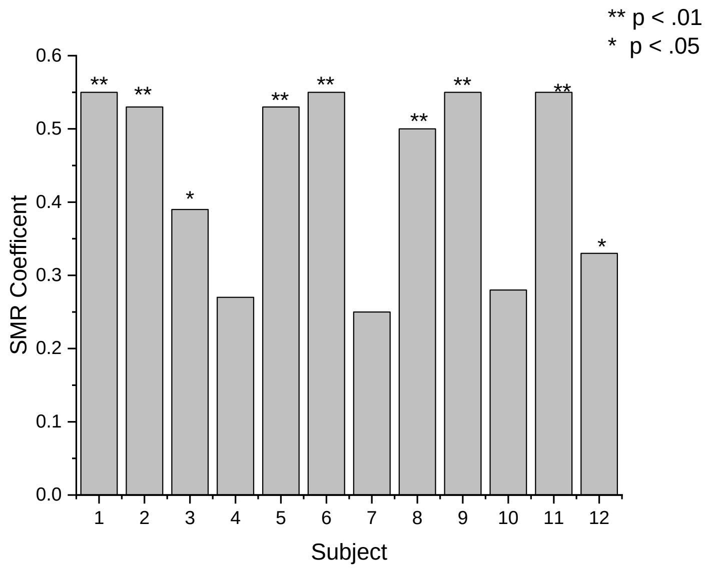

Supplementary Figure 2. Result of multilevel analysis avoidance margin ratio effect on path selection for each participant

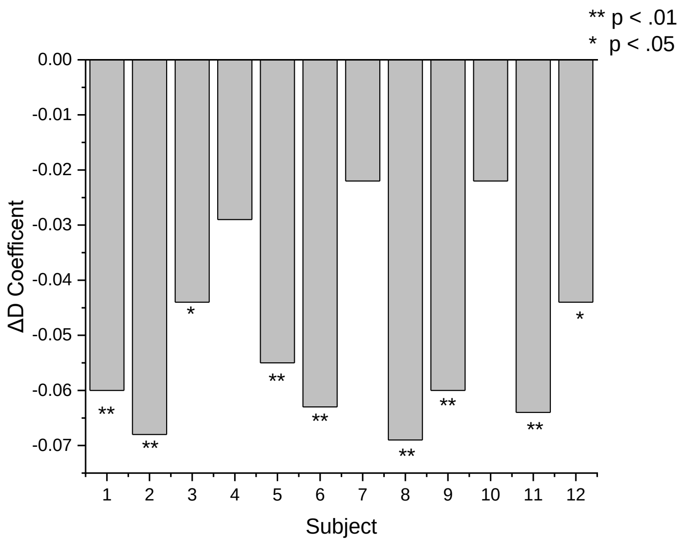

Supplementary Figure 3. Result of multilevel analysis distance to the obstacle effect on path selection for each participant

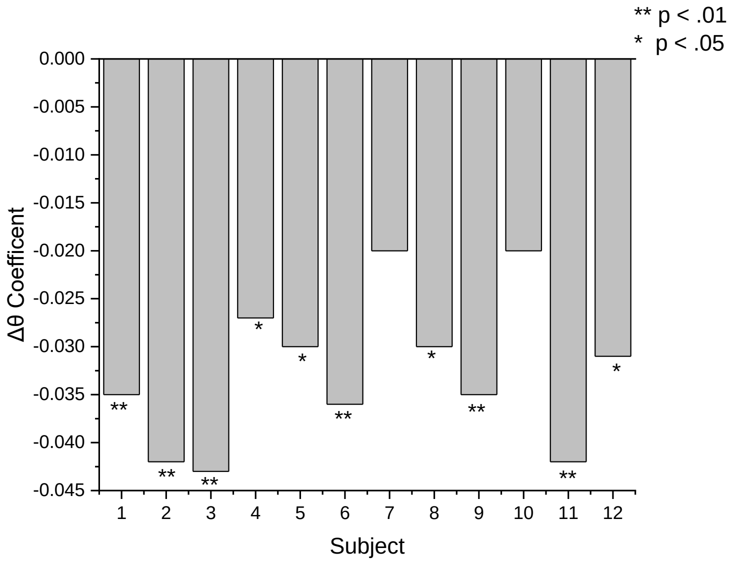

Supplementary Figure 4. Result of multilevel analysis deviation angle from the straight-line effect on path selection for each participant

(A)

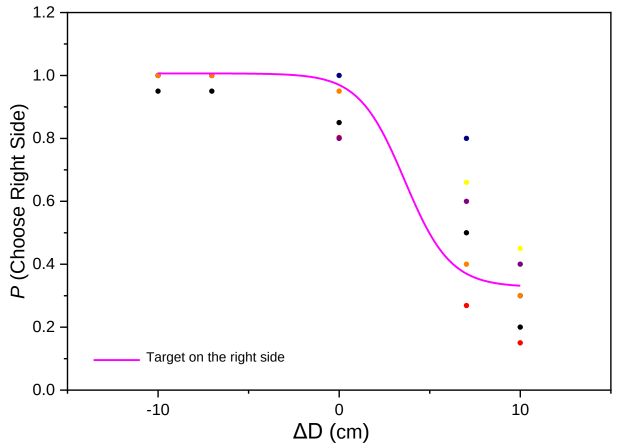
1.2
1.0
0.8
0.6
0.4
0.2
0.0
P (Choose Right Side)
Target on the right side
-10
0
10
ΔD (cm)

(B)

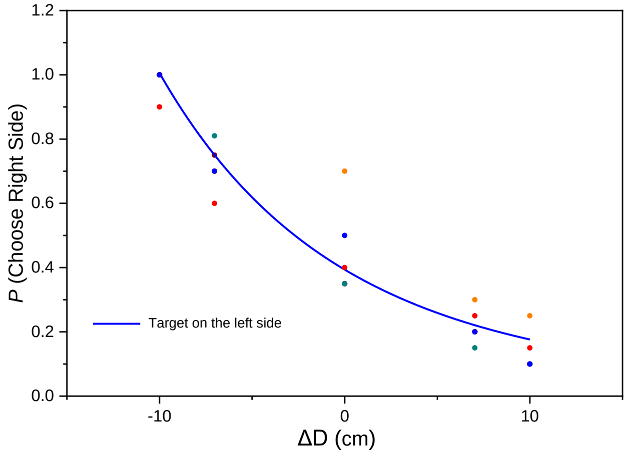
1.2
1.0
0.8
0.6
0.4
0.2
0.0
P (Choose Right Side)
Target on the left side
-10
0
10
ΔD (cm)

(C)

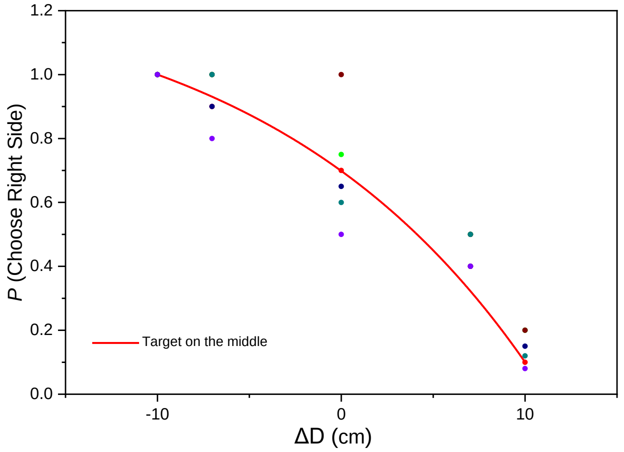
1.2
1.0
0.8
0.6
0.4
0.2
0.0
P (Choose Right Side)
Target on the middle
-10
0
10
ΔD (cm)

(D)

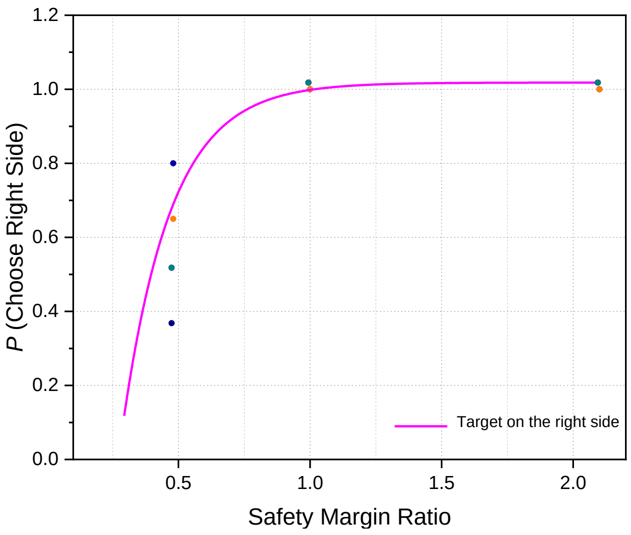
1.2
1.0
0.8
0.6
0.4
0.2
0.0
P (Choose Right Side)
0.5
1.0
1.5
2.0
Safety Margin Ratio
Target on the right side

(E)

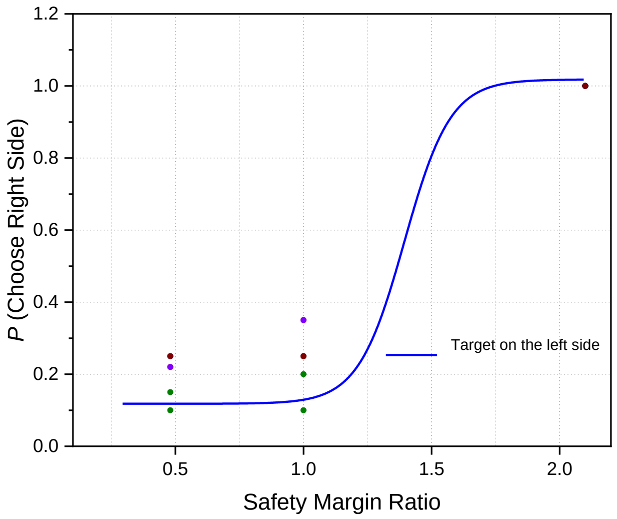
1.2
1.0
0.8
0.6
0.4
0.2
0.0
P (Choose Right Side)
0.5
1.0
1.5
2.0
Safety Margin Ratio
Target on the left side

(F)

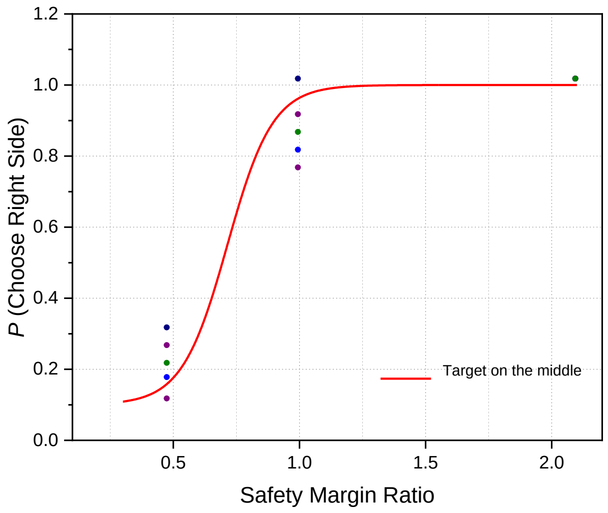
1.2
1.0
0.8
0.6
0.4
0.2
0.0
P (Choose Right Side)
0.5
1.0
1.5
2.0
Safety Margin Ratio
Target on the middle

Supplementary Figure 5. Influence of ΔD and AMR on the probability of walking to the right of the obstacle with participant data. Influence of ΔD on path selection when target was (A) on the right side, (B) on the left side, and (C) on the middle. Influence of AMR on path selection when target was (D) on the right side, (E) on the left side, and (F) on the middle

	Est.	S.E.	T val.	d.f.	P
Intercept	1.25	0.18	6.85	12.02	.001
AMR	+0.46	0.17	-2.75	12.00	.02
Δθ	-0.02	0.001	-5.27	12.28	.001
ΔD	-0.08	0.02	-3.28	11.89	.01

Supplementary Table 1. Result of multilevel analysis
